# Lessons from El Niño: gender-based violence must be on the agenda for future climate events

**DOI:** 10.1016/j.lana.2024.100820

**Published:** 2024-06-12

**Authors:** The Lancet Regional Health – Americas

April 29, 2024, was marked by one of Brazil's worst extreme weather events. The majority of cities from Rio Grande do Sul, the most populated state in southern Brazil, has been underwater, with the Guaíba river reaching its highest recorded level of 5.3 m. On May 1, 2024, the Government declared a state of public calamity, with 114 municipalities affected out of 497 at the time. As the time of writing, 160 people have died, 53 are still missing, 2.1 million were directly or indirectly affected by the floods in 469 municipalities, 77,712 were rescued, and 55,000 are currently living in shelters. The 2023–24 El Niño phenomenon highlights a heightened intensity of extreme meteorological events, which may be attributed to the escalating climate crisis. Whether the catastrophic events in Rio Grande do Sul might have been intensified by poor governance and lack of administration, the increased frequency of climate events is undeniable. The complexity of climate change events not only is an environmental problem but also has sociological consequences and presents a systemic health risk.

The adverse effects are even more noticeable for those who experience some level of vulnerability related to gender, age, race and ethnicity, and disability. Women can experience different forms of violence, such as physical violence, sexual violence, psychological violence, and economic violence. During the emergency conditions as a consequence of the severe floods in Rio Grande do Sul, there were reports of abuse against women and children in shelters, similar to those reported in other events such as Hurricane Katrina and other locations, revealing the need to address and learn from past occurrences. Strategies such as dividing shelters between sex or gender and securing a budget for specialised security during climate change events are important, but not enough.

Despite the development of national adaptation plans targeting climatic events and strategies in Latin America and Caribbean countries, climate change is accelerating and implementations should be done with haste. Specifically, gender-related supportive public policies should be on the agenda and incorporate strategies to tackle climate change and its repercussions. Although Brazil is one of the few countries in Latin America and the Caribbean with a specific climate action plan, this was published in 2016 and has not been updated since. In the plan, gender is addressed by focusing on vulnerable communities and populations, but there are no specific implementation strategies to address gender-related vulnerabilities, as observed in the lack of planning and immediate solutions during the Rio Grande do Sul floods.

A holistic and sustainable approach for planning and responding to these events is essential to preserve and protect lives and strengthen communities. The UN has released a report on conflict-related sexual violence, and one of the recommendations is to invest in services to meet the basic needs of women and girls, ensuring full and meaningful participation in the humanitarian, recovery, peace, and development processes. Amidst the chaos and despair, the Rio Grande do Sul floods also witnessed remarkable acts of bravery and compassion. Emergency responders, volunteers, and ordinary citizens displayed extraordinary courage as they risked their lives to save others. Their selfless dedication and unwavering commitment to rescue and aid those in need exemplify the resilience and strength of the human spirit in the face of adversity. However, planning for climate events should be a priority in the governmental agenda.

Disaster settings are a perfect scenario for an increase in violence, due to the combination of a disrupted social context, reduced law enforcement, and increased stressors. These stressors, such as poverty, lack of resources, and disruption in health and social services, can trigger violence—creating permissive environments for violence to occur and drivers for violence against women and girls. Gender violence is not only about sexual violence; in fact, children are also victims, as shown in a systematic review and meta-analysis focusing on the violence against children in natural disasters, wherein physical violence was reported as the most common type of violence, followed by sexual violence.

As the climate crisis escalates, the wellbeing, health, and development of populations are under threat. To prevent the exacerbation of adverse health effects, discrimination, and inequalities, we must focus on social vulnerability. The lack of preventive actions and protective systems aggravates post-disaster contexts. Actions such as violence prevention strategies, protection measures for women and children, and their inclusion in planning and policies ought to be addressed. Communities need to be resilient in responding immediately to the climate crisis, which can be achieved with the revision of urban planning and managing disasters in the region. As new extreme events occur, we must put plans into action. Time is of the essence.

